# Online versus classroom teaching for medical students during COVID-19: measuring effectiveness and satisfaction

**DOI:** 10.1186/s12909-021-02888-1

**Published:** 2021-08-28

**Authors:** Abdullh AlQhtani, Nasser AlSwedan, Abdullelah Almulhim, Raghad Aladwan, Yara Alessa, Kholoud AlQhtani, Malak Albogami, Khalid Altwairqi, Fahad Alotaibi, Abdulmajeed AlHadlaq, Osama Aldhafian

**Affiliations:** 1 College of Medicine, Prince Sattam Bin Abdulaziz University, Al-Kharj, Saudi Arabia; 2 College of Medicine, Imam Mohammad Ibn Saud Islamic University, Riyadh, Saudi Arabia; 3 College of Medicine, Qassim University, Buraydah, Saudi Arabia; 4 College of Medicine, Princess Nourah Bint Abdulrahman University, Riyadh, Saudi Arabia; 5 College of Medicine, Umm Al-Qura University, Mecca, Saudi Arabia; 6 College of Medicine, Imam Abdulrahman Bin Faisal University, Dammam, Saudi Arabia

**Keywords:** COVID-19, E-Learning, Medical students, Effectiveness, Satisfaction

## Abstract

**Background:**

The COVID-19 pandemic and physical distancing have had a significant impact on the conversion of traditional teaching methods to online teaching methods, which although not uncommon in medical schools, has to date only been used for some aspects of the teaching process. Thus, we aimed to measure the effectiveness of e-learning during the COVID-19 pandemic, as well as medical students’ preferences regarding e-learning and classroom teaching, and the possibility of applying it post-pandemic.

**Methods:**

A cross-sectional online survey of medical students (N = 376) in six medical schools was carried out after their second semester, from August 15 to 20, 2020. Ten parameters were measured for the effectiveness of e-learning based on a 5-point Likert-scale and five parameters were measured for satisfaction.

**Results:**

e-learning was more or equally effective in four parameters such as assignment submission and meeting individual needs, but less effective in six parameters, including building skills and knowledge, and interaction level. Satisfaction was either high or neutral in all five parameters.

**Conclusions:**

Our findings have shown that e-learning can assist the teaching process in medical schools in some respects, but cannot be used for the entire teaching process.

## Background

COVID-19 is an emerging disease caused by the severe acute respiratory syndrome coronavirus (SARS-CoV-2) that causes illnesses ranging from the common cold to more severe diseases, and patients may experience pneumonia and abdominal distress with other functional failures [1]. It emerged at the end of 2019 and was first reported in China as an unknown pneumonia [2]. Thereafter, the disease spread worldwide, leading to the World Health Organization declaring it a pandemic [3]. Despite unprecedented attempts to restrain the disease, COVID-19 has at the time of writing infected 41 million people and caused the deaths of more than half a million globally [4]. The spread of COVID-19 is dramatically increasing due to social mixing and research has proven that physical distancing has a significant impact on limiting its spread [2]. As a result, most governments have imposed quarantines to contain COVID-19 and are implementing all possible activities online, including educational processes in institutions, office work, and a wide range of other activities [3, 4]. In Saudi Arabia, the government suspended physical attendance at workplaces in all government and private agencies, implemented online services, and activated a remote education system in the education sector.

Over the last decade, educational resources have rapidly expanded for undergraduate medical students. Presently it comprises both traditional and online (or e-learning) tools, which include textbooks, lectures, and tutorials [5, 6]. This combination of methods is now a well-established concept, known as “Blended learning [7].

Although a meta-analysis indicated that e-Learning is associated with positive outcomes [8], some studies have shown that there are numerous barriers to the implementation of e-Learning in medical schools e.g., time constraints, poor technical skills, inadequate infrastructure, absence of institutional strategies and support [9]. In this study we aimed to evaluate the (a) effectiveness of online classes and students’ level of satisfaction in terms of gaining knowledge, (b) the balance between practical and theoretical experiences, (c) and availability of e-resources.

## Methods

### Design

A cross-sectional online survey was conducted among medical students by email, after approval was obtained from the institutional review board.

### Participants

900 students were invited to take part in the study and 376 responded (male: 86.4 %; female: 13.6 %; average age and standard deviation: 22.9 ± 2.34 years) from 1st to 6th year in six medical schools in the Riyadh region of Saudi Arabia. All the medical schools are using the active eLearning (live lectures online with discussion, Problem-Based Learning (PBL), Integrated Clinical Case Discussion (ICCD), Tutorial, and Self-Directed Learning. They consented to voluntarily participate in the survey that was carried out from August 15 to 20, 2020 after completion of their academic year.

### Materials

The survey was based on the effectiveness of learning through e-classes and satisfaction levels. it was designed based on a 5-point Likert-scale and developed by Kaur et al. [10] the same survey was used and was piloted on 10 students. Satisfaction with various aspects of online learning was assessed using five Likert-scale items ranging from 1 (strongly disagree) to 5 (strongly agree) [14]. Similarly, convenience was assessed using ten Likert-scale items ranging from 1 (much less effective) to 5 (much more effective).

### Statistical analysis

Statistical analysis was performed using R v 3.6.3. (R: The R Project for Statistical Computing (r-project.org) Counts and percentages were used to summarize survey responses while mean and standard deviation were used to summarize the central tendency and distribution of continuous variables, respectively. An average satisfaction score was calculated for each respondent. Linear regression was used to assess the association between demographic characteristics and satisfaction with online classes. One-way ANOVA with post-hoc pairwise comparisons was used to assess whether satisfaction was significantly different based on medical school and year of study. Pearson’s correlation was used to assess the association between convenience of online learning and satisfaction with online learning. Previously defined cut-off points were used to interpret Pearson’s correlation coefficient [11]. Hypothesis testing was performed at 5 % level of significance.

## Results

More than half of the respondents were from Prince Sattam bin AbdulAziz University (56.6 %), while 17.6 and 8.78 % were from Al-Maarefa and Imam Muhammed Ibn Saud Universities, respectively (Table 1). Three quarters (*N* = 282, 75 %) of the respondents had not attended any online medical classes before the pandemic.

**Table. 1:** Descriptive statistics for the study sample

	*N *= 376
**Gender**:	
Male	325 (86.4 %)
**Age**	22.9 (2.34)
**Medical school**:	
Alfaisal University	24 (6.38 %)
Almaarefa University	66 (17.6 %)
Imam Muhammad ibn Saud University	33 (8.78 %)
King Saud University	25 (6.65 %)
King Saud University for Health Sciences	15 (3.99 %)
Prince Sattam bin Abdulaziz University	213 (56.6 %)
**Year in medical school**:	
1st year	47 (12.5 %)
2nd year	53 (14.1 %)
3rd year	42 (11.2 %)
4th year	64 (17.0 %)
5th year	58 (15.4 %)
6th year	112 (29.8 %)
**Online medical classes before this pandemic**:	
No	282 (75.0 %)
Yes	94 (25.0 %)

**Fig. 1:**
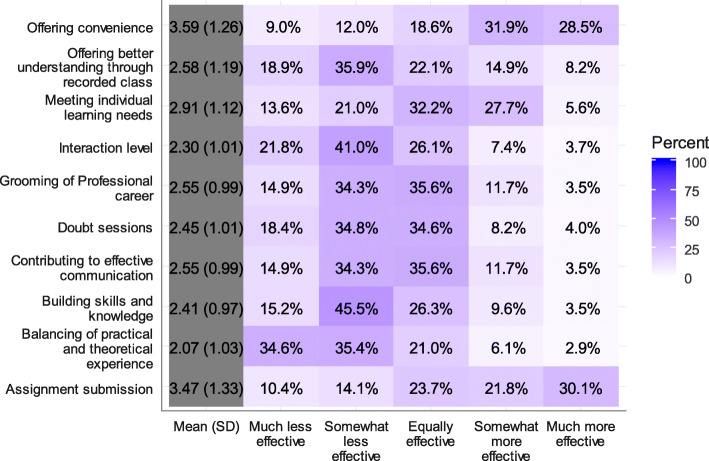
Effectiveness of online learning compared to regular classroom settings

Online learning was more convenient, as demonstrated by the fact that 28.5 and 31.9 % of the respondents thought that it was much more effective and somewhat more effective, respectively (Fig. 1).

In terms of balancing practical and theoretical experiences, the majority of students found online learning to be either much less effective (34.6 %) or somewhat less effective (35.4 %). Respondents thought that assignment submission was either much more effective (30.1 %), somewhat more effective (21.8 %), or equally effective (23.7 %) compared to regular classroom teaching. Slightly less than half of the respondents (45.5 %) thought that online learning was less effective in building skills and knowledge and 41 % thought that the interaction level was somewhat less effective.

**Fig. 2:**
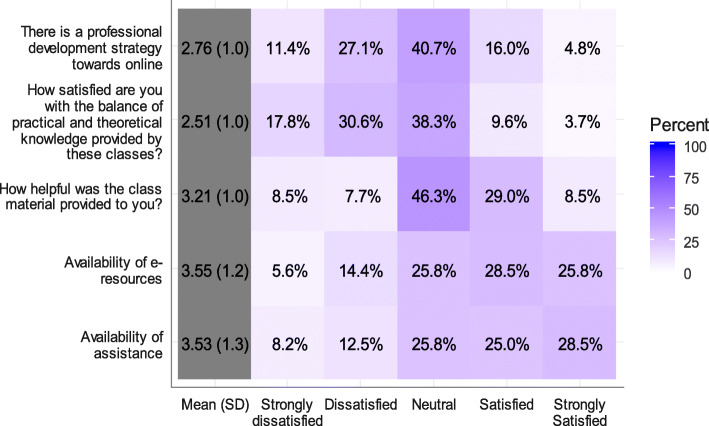
Satisfaction with online classes

Results showed a somewhat negative attitude towards the effectiveness of online learning in grooming students’ professional career, contributing to effective communication, and organizing doubt sessions (Question and Answer session). Regarding individual learning needs, 27.7 % of the respondents thought that online learning was somewhat more effective in meeting individual learning needs, while 32.2 % thought that it was equally effective compared to classroom settings. The average satisfaction score was highest for assignment submission (3.47 ± 1.33) and lowest for balancing practical and theoretical experience (2.07 ± 1.03) (Fig. 2).

**Fig. 3:**
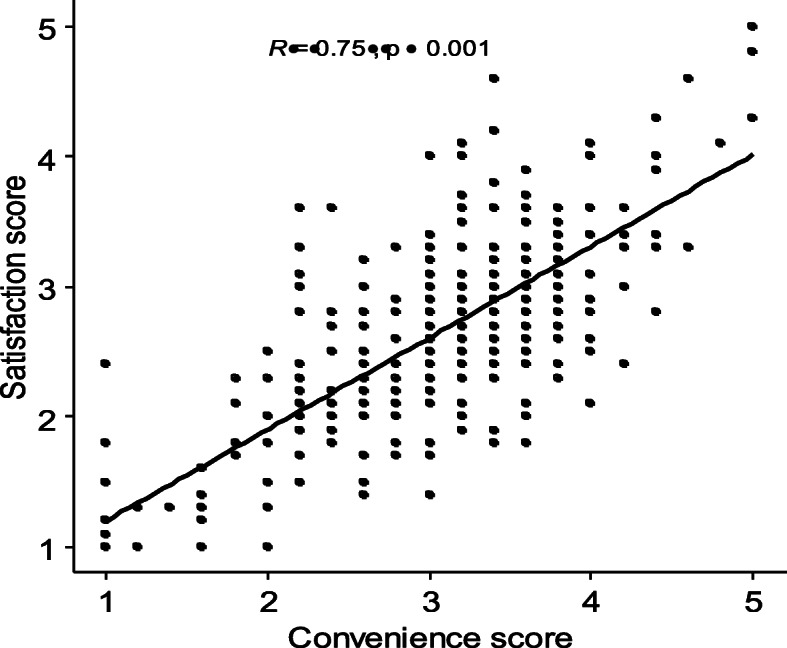
Correlation between convenience and satisfaction

Half of the students were satisfied with the availability of assistance (28.5 and 25 % were strongly satisfied and satisfied, respectively) and resources (25.8 and 28.5 % were strongly satisfied and satisfied, respectively) (Fig. 3).

Approximately half of the students (46.3 %) were neither satisfied nor dissatisfied with the help provided through class materials and 40.3 % were neutral regarding the professional development strategy towards online classes. Satisfaction was highest for the availability of e-resources (3.55 ± 1.2) and assistance (3.53 ± 1.3) and lowest for the balance between practical and theoretical knowledge (2.51 ± 1) (Table 2).

**Table 2:** Factors associated with satisfaction score

	Sat		
***Predictors***	***Estimates***	***95 % CI***	***p***
**Gender**	Ref		
Female	0.38	0.15–0.61	**0.001**
Male	-0.05	-0.09 – -0.02	**0.002**
**Age**			
**Online medical classes before pandemic?**			
No			
Yes	0.05	-0.13–0.23	0.586

Linear regression analysis showed that the average satisfaction score was significantly higher for males compared to females (B = 0.38, *p* = .001) which indicates that the average satisfaction score was higher by 0.38 points for males compared to females. Higher age was associated with lower satisfaction with online learning (B = -0.05, *p* < .05). Receiving online medical classes before the pandemic was not associated with satisfaction with online learning (B = 0.05, *p* > .05).

Results showed a high positive correlation between convenience and satisfaction with online learning (r = .75, *p* < .001) which indicates that students who perceived online learning as convenient, were more likely to be satisfied.

**Fig. 4:**
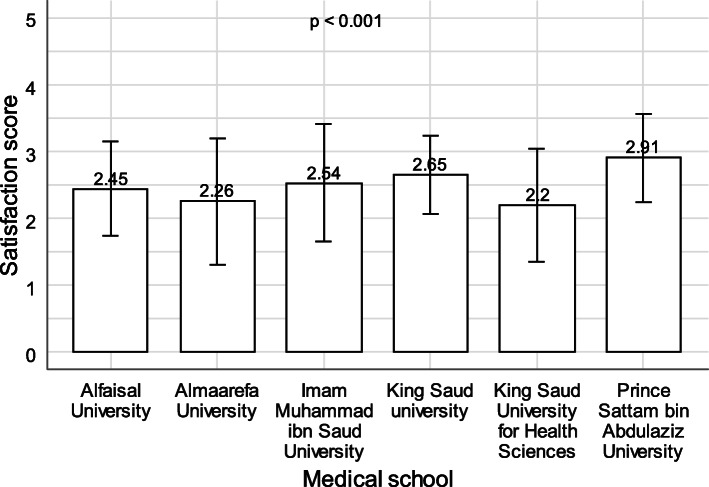
Association between medical school and satisfaction with online learning

One-way ANOVA showed that there was a statistically significant association between medical school and satisfaction with online learning (*p* < .001). The average satisfaction score was significantly lower in students from Al-Maarefa University, Al-Faisal University, and King Saud University for Health Sciences compared to students from Prince Sattam Bin AbdulAziz University (*p* < .05). None of the remaining comparisons were statistically significant (Fig. 4).

**Fig. 5:**
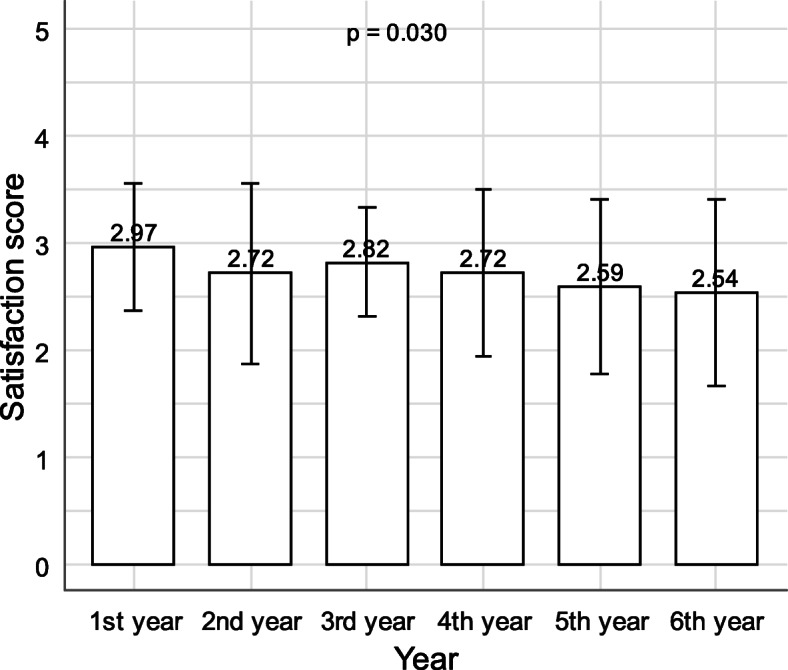
Association between school year and satisfaction with online learning

Moreover, results showed a statistically significant linear trend in the association between medical school year and satisfaction with online learning (*p* < .001). The scores show that satisfaction tends to decrease with the increase in the medical school year (Fig. 5).

## Discussion

Traditional teaching methods (including mentoring, face-to-face contact, and supervision) play an important role in the development of higher-order cognitive skills [12], and apart from face to face contact, interaction and discussion are also currently among the best way for students to learn these important skills. However, e-learning will assume an important role in the teaching of medical students in the future [13]. Online resources for medical students’ educational materials (including lectures, textbooks and tutorials) have expanded rapidly and mobile technology and online tools for learning are increasingly accessible [5, 6]. However, studies show that there are many significant barriers to the adoption and implementation of e-learning by medical schools [9].

The COVID-19 pandemic has accelerated the implementation of e-learning and most medical schools used it regardless of their readiness. In our study, we tried to establish the best way to use e-learning and further establish whether it can be used post-pandemic.

On the positive side, we found that 65.5 % of the respondents thought that e-learning is more or equally effective for meeting individual learning needs than traditional teaching methods, which is an opposite finding to that of Kaur et al. [10]. Regarding online assignment submission, 51.9 % of the respondents thought that it was much more or somewhat more effective, while 23.7 % thought it was equally effective compared to regular classroom submission. This is positive feedback that can be applied in the future, retaining assignment submission and homework completion as online activities. Regarding resources, more than half of the respondents were satisfied with the availability of online assistance and easy access to resources for tutors and students. This suggests that continuing some aspects of online teaching such as assignment submission and online teaching support post-pandemic, may be beneficial.

On the negative side, more than half of the respondents thought that online learning was much or somewhat less effective in balancing practical and theoretical experience. Satisfaction tends to decrease with increasing years of study, especially when the practical aspects of teaching are at their peak. This is due to the fact that at this stage of study, it is important for students to have contact with and inspect actual patients and be able to recognize various symptoms face-to-face. These processes are crucial for students’ success to ensure that they will be good doctors in the future [13].

Additionally, slightly less than half of the respondents thought that online learning was less effective in building skills and knowledge. Moreover, 41 % thought that the interaction level was somewhat less effective. Literature shows that high levels of satisfaction are related to well-structured and organized e-courses which also have a greater impact on knowledge accumulation and student performance compared with traditional learning [14].

In the literature, Vallée A et al. in his recent systematic review found that blended learning has better effects on knowledge outcomes compared to traditional learning [15]. In a systematic review, Wilcha RJ et al. found online teaching during the COVID pandemic to be effective and that educational institutions are working to improve their virtual teaching resources [16]. Dost S et al. anticipate more application of online teaching methods within traditional medical education [17].

Regarding the limitations of our study, we had hoped to obtain a higher number of participants and the response rate from some universities was low. Additionally, the parameters used in the questionnaire have been previously used in only one study and may need some improvement in future studies. We think that each parameter should be studied individually, enhancing the scope for future research to improve the quality of medical teaching.

## Conclusions

Combining the advantages of e-learning and traditional teaching methods for improving medical teaching and student experience is the best and most practical way to maintain or even advance the level of teaching. This is called “blended teaching” as supported by Dodiya et al. [18]. To build on the results of our study, future research should focus on analysing each teaching method (including lectures, practical sessions) separately to indicate which teaching method (e-learning or traditional) is more preferable and how maximum benefit can be derived from each approach.

## Data Availability

The data that support the findings of this study are available from the corresponding author, upon request.
